# Can the low and high b-value distribution influence the pseudodiffusion parameter derived from IVIM DWI in normal brain?

**DOI:** 10.1186/s12880-020-0419-0

**Published:** 2020-02-10

**Authors:** Yu-Chuan Hu, Lin-Feng Yan, Yu Han, Shi-Jun Duan, Qian Sun, Gang-Feng Li, Wen Wang, Xiao-Cheng Wei, Dan-Dan Zheng, Guang-Bin Cui

**Affiliations:** 1 Department of Radiology and Functional and Molecular Imaging Key Lab of Shaanxi Province, Tangdu Hospital, Air Force Medical University (Fourth Military Medical University), Xi’an, 710038 Shaanxi People’s Republic of China; 2 MR Research China, GE Healthcare China, Beijing, 100176 China

**Keywords:** Intravoxel incoherent motion, B-value, Pseudodiffusion, Brain, Diffusion weighted imaging, Number of excitation

## Abstract

**Background:**

Our study aims to reveal whether the low b-values distribution, high b-values upper limit, and the number of excitation (NEX) influence the accuracy of the intravoxel incoherent motion (IVIM) parameter derived from multi-b-value diffusion-weighted imaging (DWI) in the brain.

**Methods:**

This prospective study was approved by the local Ethics Committee and informed consent was obtained from each participant. The five consecutive multi-b DWI with different b-value protocols (0–3500 s/mm^2^) were performed in 22 male healthy volunteers on a 3.0-T MRI system. The IVIM parameters from normal white matter (WM) and gray matter (GM) including slow diffusion coefficient (D), fast perfusion coefficient (D*) and perfusion fraction (f) were compared for differences among defined groups with different IVIM protocols by one-way ANOVA.

**Results:**

The D* and f value of WM or GM in groups with less low b-values distribution (less than or equal to 5 b-values) were significantly lower than ones in any other group with more low b-values distribution (all *P* <  0.05), but no significant differences among groups with more low b-values distribution (*P* > 0.05). In addition, no significant differences in the D, D* and f value of WM or GM were found between group with one and more NEX of low b-values distribution (all *P* > 0.05). IVIM parameters in normal WM and GM strongly depended on the choice of the high b-value upper limit.

**Conclusions:**

Metrics of IVIM parameters can be affected by low and high b value distribution. Eight low b-values distribution with high b-value upper limit of 800–1000 s/mm^2^ may be the relatively proper set when performing brain IVIM studies.

## Background

As an advanced magnetic resonance imaging (MRI) technique, diffusion-weighted imaging (DWI) is considered the one of the most sensitive pulse sequences for early pathological changes. However, previous studies have shown that perfusion can substantially confound diffusion measurements because of the incoherent motion of blood in pseudorandom capillary networks at the macroscopic level, and have proposed intravoxel incoherent motion (IVIM) imaging to measure microvascular perfusion [1]. For its ability of utilizing biexponential model to extract the perfusion-related information from a diffusion sequence [1], IVIM DWI has been successfully applied to clinical research with the great improvement of gradient coil systems for diffusion MR imaging over the recent years, especially in the body or head and neck [2–11]. Perfusion has been proven an important surrogate marker of many physiologic or pathologic processes as well as a predictor of recovery with reperfusion in patients of acute ischemic stroke [12]. Also, perfusion measurement has demonstrated improved sensitivity and predictive value for tumor grading and prognosis [13–19].

The IVIM model is a two-compartment model and includes terms for the fraction of received signal attributed to moving blood (fractional perfusion, f), the diffusion caused by moving blood (pseudodiffusion, D*), and a diffusion component free of perfusion effects (true molecular diffusion, D). The model requires the collection of both low and high b-values as the perfusion effect becomes largely negligible as the b-value is increased beyond approximately 200 s/mm^2^ [1, 20, 21].

Previous studies have supported that IVIM DWI could be potentially useful in measuring brain perfusion [22–30]. However, the calculated perfusion-related IVIM maps usually suffer from low image quality and high parametric variance [31]. Many factors might influence the accuracy of pseudodiffusion parameter derived from IVIM DWI, such as the low b-values distribution, number of excitation (NEX), repetition time (TR), echo time (TE), field strength, and diffusion preparation pulses [32–36]. To date, the b value distributions are chosen heuristically and vary greatly among researchers.

In spite of an increasing number of applications of IVIM DWI, there is no clear consensus regarding the optimal protocol that should be used. Concerning the used acquisition parameters, the amount and distribution of b values varied greatly. For example, IVIM studies in the glioma grading have used different b-value distributions and obtained different trend values for IVIM parameters [22, 37–40]. Specifically, using 13 b values (0–1000 s/mm^2^), Togao and his colleagues found significantly lower D and higher f in high grade glioma (HGG) than in low grade glioma (LGG), while no statistically difference of D* in two groups [37]. In another research, in which 20 b values were used (0–3500 s/mm^2^), reported a significant higher D* was found in HGG group [38]. Recent studies have shown pseudodiffusion in the liver tended to be underestimated when too few low b-values (0 < b < 50 s/mm^2^) were included in the distribution [41]. However, to our knowledge, the optimal b-value distribution for IVIM DWI of the brain is scarce.

Ideally speaking, dense sampling of b-values would certainly guarantee quantification data quality and repeatability. However, more b-value samples involve longer scan time, making the measurement less practical and vulnerable to subject motion. We hypothesize that the amount of b-values could be reduced while still enabling correct IVIM parameter estimation, without affecting the reproducibility of the technique.

Our study aims to reveal whether the low b-values distribution, high b-values upper limit, and NEX for low b-values influence the accuracy of IVIM parameter in the brain, and to determine the minimal amount and optimal b-value distribution necessary for reproducible brain IVIM parameter quantification.

## Methods

### Subjects

**Fig. 1:**
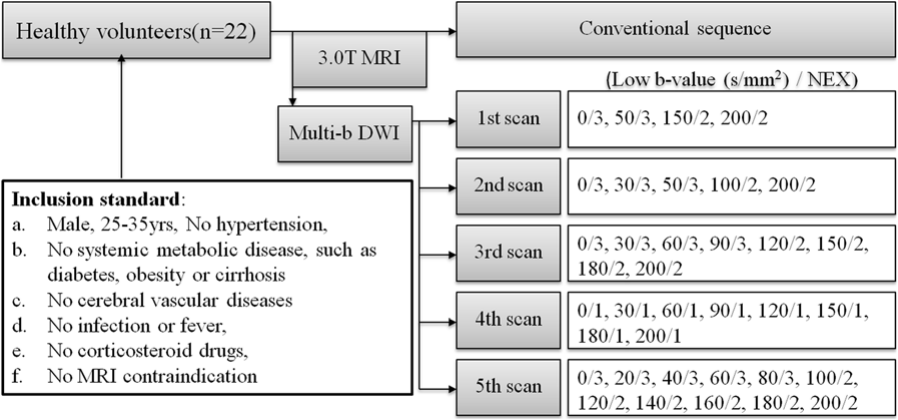
Flow diagram shows subjects selection process and multi-b-value DWI protocol. Note: Each set of low b-values was in addition to a high b-value distribution of 300, 500, 800, 1000, 1500, 2000, 3000 and 3500 s/mm^2^, with NEX of 2,2,2,2,2,4,4 and 6, respectively

Between November and December 2014, 22 male healthy volunteers were recruited (mean age, 28 years; range, 25–34) with inclusion standard as follows: no potential vascular risk factors or diseases affecting brain microvascular perfusion including hypertension or cerebral vascular diseases, systemic metabolic disease, such as diabetes, obesity or cirrhosis; no infection or fever, and use of corticosteroid drugs; no MRI contraindication and appearing normal on conventional brain MR image (as shown in Fig. 1).

### Brain MR image data acquisition

The whole brain MRI examinations were performed on a 3.0-T MRI system (Discovery MR750, GE Healthcare, Milwaukee, WI, USA) with a 40-mT/m maximum gradient capability and an eight-channel head coil (GE Medical Systems). Conventional MRI sequence and five consecutive multiple b-value DWI sequences of brain were performed during one examination.

Conventional MRI sequences included T1-weighted spin echo in the transverse plane (TR/TE, 1750 ms/24 ms; matrix size, 256 × 256; field of view (FOV), 24 cm × 24 cm; NEX, 1; slice thickness, 5 mm; gap, 1.5 mm), T2-weighted fast spin echo in the transverse planes (TR/TE, 4247 ms/93 ms; matrix size, 512 × 512; FOV, 24 cm × 24 cm; NEX, 1; slice thickness, 5 mm; gap, 1.5 mm) and sagittal planes (TR/TE, 10,639 ms/96 ms; matrix size, 384 × 384; FOV, 24 cm × 24 cm; NEX, 2; slice thickness, 5 mm; gap, 1.0 mm), and fluid-attenuated inversion recovery (FLAIR) in the transverse plane (TR/TE, 8000 ms/165 ms; matrix size, 256 × 256; FOV, 24 cm × 24 cm; NEX, 1; slice thickness, 5 mm; gap, 1.5 mm) [22].

Five consecutive multi-b-value DWI sequences of brain were performed after the conventional MRI. The details of five different parameter sets of low b-value distribution and NEX were showed in Fig. 1. Different parameter sets of b values were applied with a single-shot diffusion-weighted spin-echo echo-planar sequence. Parallel imaging was used with an acceleration factor of 2. A local shim box covering the whole brain was applied to minimize susceptibility artifacts. In total, 20 axial slices covering the entire brain were obtained with a 24 cm × 24 cm FOV, 5 mm slice thickness, 1.5 mm slice gap, 3000 ms TR, Minimum TE, 128 × 128 matrix and one diffusion preparation pulses [22].

### IVIM DWI data processing

All data were analyzed and processed on a GE ADW4.6 workstation (MR750, GE Healthcare, Milwaukee, WI, USA), using the MADC program of Functool software. The mean IVIM parameters were measured independently by one experienced radiologist (Y.-C.,H, with 12 years of experience in radiology).

According to IVIM theory, the relationship between signal intensity and b values can be expressed based on an Eq. (1) [22]:

1 $$ {\mathrm{S}}_{\mathrm{b}}/{\mathrm{S}}_0=\mathrm{f}\ \exp \left(-\mathrm{b}\ \mathrm{D}\ast \right)+\left(1-\mathrm{f}\right)\exp \left(-\mathrm{b}\ \mathrm{D}\right) $$

where S_0_ = signal intensity at the b value of 0 s/mm^2^; S_b_ = signal intensity at the b value denoted by the subscript; D is the slow diffusion component that reflects random motion of intra- and intercellular water molecules; f is the fraction of the diffusion linked to microcirculation, and D* is the fast diffusion component representing incoherent microcirculation within the voxel. D* is usually expected to be at least one order of magnitude higher than D [2, 22, 42], the influence of D* on signal decay can be neglected for b values greater than 200 s/mm^2^. Eq. (1) can then be simplified, and the estimation of D can be obtained by using only b values greater than 200 s/mm^2^ with a simple linear fit eq. (2) [22, 43]:

2 $$ {\mathrm{S}}_{\mathrm{b}}/{\mathrm{S}}_0=\left(1-\mathrm{f}\right)\exp \left(-\mathrm{b}\ \mathrm{D}\right) $$

**Fig. 2:**
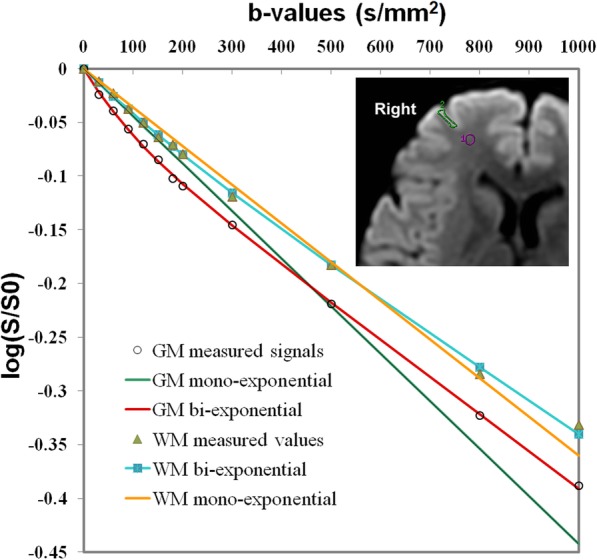
Comparison of the bi- and mono-exponential fitting of the diffusion signal decay over a wide-range of b-values (up to 1000 s/mm^2^) in normal brain, with more obvious pseudodiffusion effect in GM than in WM. Note: Axial diffusion-weighted trace image (b = 1000 s/mm^2^) shows ROIs placed in right frontal white matter (WM) and gray matter (GM)

**Fig. 3:**
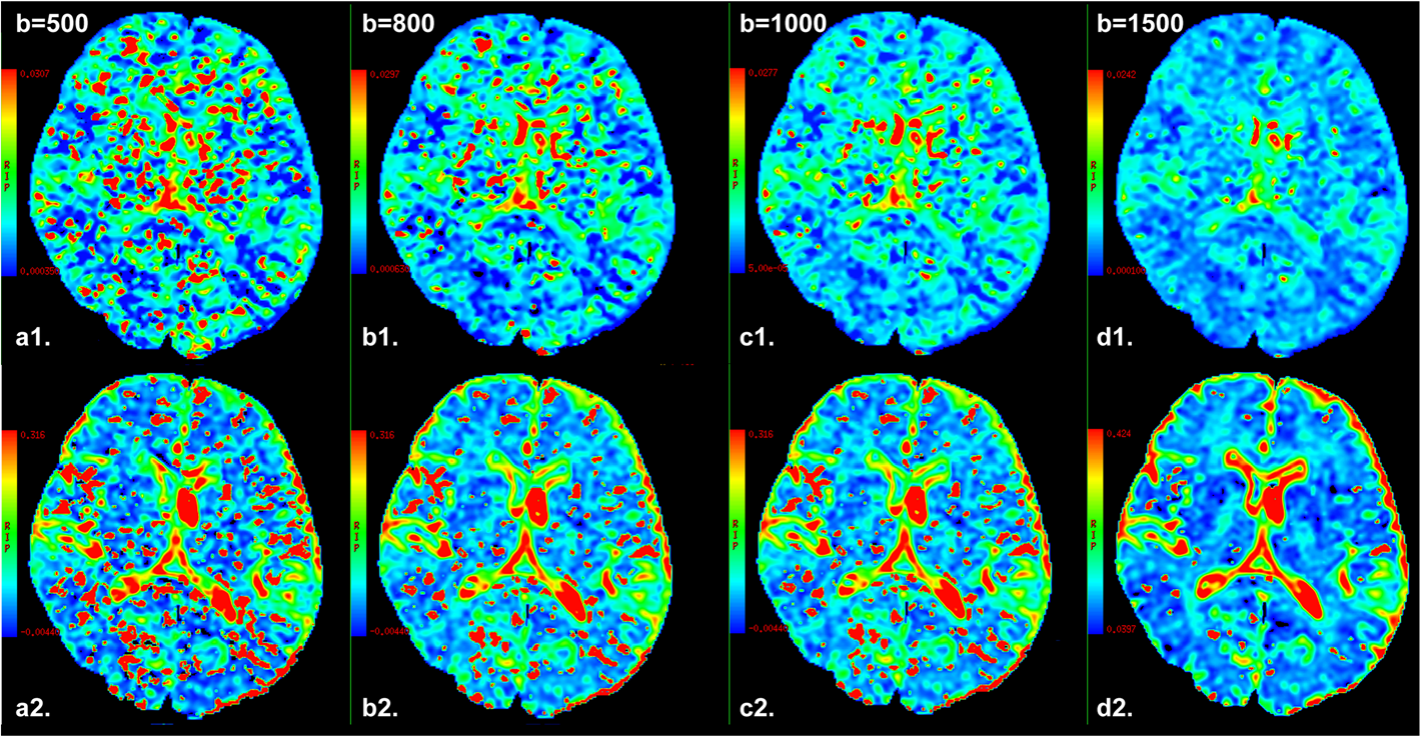
Brain pseudodiffusion parametric maps from one representative subject. The top row depicts D* maps (a1-d1) calculated by 4 sets of multiple b-value DWI with different high b-value upper limit (500, 800, 1000, 1500 s/mm^2^, respectively) and the same low b-value distribution (0, 30, 60, 90, 120, 150, 180, 200 s/mm^2^), meanwhile the bottom row shows corresponding f maps (a2-d2) derived from IVIM DWI model in normal brain. As the increase of high b-value upper limit, the red color area representing fast diffusion effect in brain tissue was reduced on pseudodiffusion images, reflecting increased fast diffusion effect. At the same time, much red color area was displayed at 500 s/mm^2^ high b-value upper limit (a1 or a2), and relative absent red area on pseudodiffusion images when high b-value upper limit reached 1500 s/mm^2^ (d1 or d2)

The D, D* and f values were calculated according to the bi-exponential model with eq. (1) and (2). First, for high b values (b > 200 s/mm^2^), S_b_ was first fitted to eq. (2) using a linear model, and the D value was calculated. In a second step, the f and D* values were calculated by using a nonlinear regression algorithm according to eq. (1), while keeping D constant. Freehand region of interests (ROIs) were placed in right frontal white matter (WM) and gray matter (GM) (as shown in Fig. 2). The mean ROI area was range from 25 to 35 mm^2^. According to the bi-exponential fitting of the diffusion signal decay over different sets of b values (as shown in Fig. 2 and Table 1), the IVIM parameter maps were generated based on the D, D* and f values derived from Eq. (1) and (2) (as shown in Fig. 3), and the mean D, D*, and f values in the corresponding ROIs were obtained, respectively.

**Table 1:** Defined groups based on IVIM DWI protocol with different b-value distribution in brain

Group	Low b value distribution (s/mm^2^)	High b value distribution (s/mm^2^)
L1	0, 50, 150, 200	same high b value distribution: 300, 500, 800, 1000 with NEX of 2,2,2 and 2, respectively
L2	0, 30, 50, 100, 200	
L3	0, 30, 60, 90, 120, 150, 180, 200	
L3_1	0, 30, 60, 90, 120, 150, 180, 200	
L4	0, 20, 40, 60, 80, 100, 120, 140, 160, 180, 200	
L3_L	0, 30, 60, 90, 120, 150, 180, 200	
H1	same low b value distribution: 0, 30, 60, 90, 120, 150, 180, 200 with NEX of 2,2,2,2,2,4,4 and 6, respectively	300, 500, 800
H2		300, 500, 800, 1000
H3		300, 500, 800, 1000, 1500
H4		300, 500, 800, 1000, 1500, 2000
H5		300, 500, 800, 1000, 1500, 2000, 3000
H6		300, 500, 800, 1000, 1500, 2000, 3000, 3500

Group L3_1: Low b value distribution with a NEX of 1 for each low b-value. Group L3_L: Obtain the IVIM parameters by placing the ROI in left frontal lobe based on the data of Group L3

The monoexponential fitting of the diffusion signal decay in WM and GM ROIs (as shown in Fig. 2) was conducted by using the following eq. (3) [44]:

3 $$ {\mathrm{S}}_{\mathrm{b}}/{\mathrm{S}}_0=\exp \left(-\mathrm{b}\times \mathrm{ADC}\right) $$

where S_b_ is the signal intensity in the pixel with diffusion gradient b (all acquired b values), and S_0_ is the signal intensity in the pixel without diffusion gradient.

### Group definition

Table 1 summarizes the defined groups based on IVIM DWI protocol with different b-value distribution. Firstly, we determine the L1 to L4 groups to explore the effect of low b-value distribution on IVIM values, by keeping the high b values (300, 500, 800, and 1000 s/mm^2^) and changing the amount of low b values with same NEX of 2 for each small b value [45]: group L1 (low b value: 0, 50, 150 and 200 s/mm^2^), L2 (low b value: 0, 30, 50, 100 and 200 s/mm^2^), L3 (low b value: 0, 30, 60, 90,120,150,180 and 200 s/mm^2^), and L4 (low b value: 0, 20, 40, 60, 80, 100, 120, 140, 160, 180 and 200 s/mm^2^). Secondly, for revealing the effect of NEX on the IVIM parameters, we determine the group L3–1 (low b-value distribution are same with group L3, but with a small NEX of 1 for each low b-values). In order to validate the consistency of IVIM processing, we define the group L3-L (b-value distribution and NEX are completely same with group L3) by placing the ROI in left frontal lobe during data processing. In addition, we try to explore the effect of high b-values upper limit on IVIM metrics, and define group H1 (high b value: 300, 500 and 800 s/mm^2^), H2 (high b value: 300, 500, 800 and 1000 s/mm^2^), H3 (high b value: 300, 500, 800, 1000 and 1500 s/mm^2^), H4 (high b value: 300, 500, 800, 1000, 1500 and 2000 s/mm^2^), H5 (high b value: 300, 500, 800, 1000, 1500, 2000 and 3000 s/mm^2^) and H6 (high b value: 300, 500, 800, 1000, 1500, 2000, 3000 and 3500 s/mm^2^) based on the different high b-value distribution, with a same set of low b value (0, 30, 60, 90, 120, 150, 180 and 200 s/mm^2^).

### Statistical analysis

All statistical analyses were performed with IBM SPSS 20.0 software (IBM Corp, Chicago, IL, USA). Numerical variables were denoted as the mean and standard deviation. D, D* and f in WM or GM were tested for differences among group L1, L2, L3, L3–1, L4 and L3-L, and among group H1, H2, H3, H4, H5 and H6 by one-way ANOVA, and further post hoc multiple comparisons were performed with Bonferroni test (equal variances assumed) and Dunett’s T3 test (equal variances not assumed). D* and f values were compared for the differences between the WM and GM by using independent sample t test. *P* <  0.05 was considered statistically significant.

## Results

### Pseudodiffusion parameter of WM or GM might be underestimated using less low b-values distribution

**Fig. 4:**
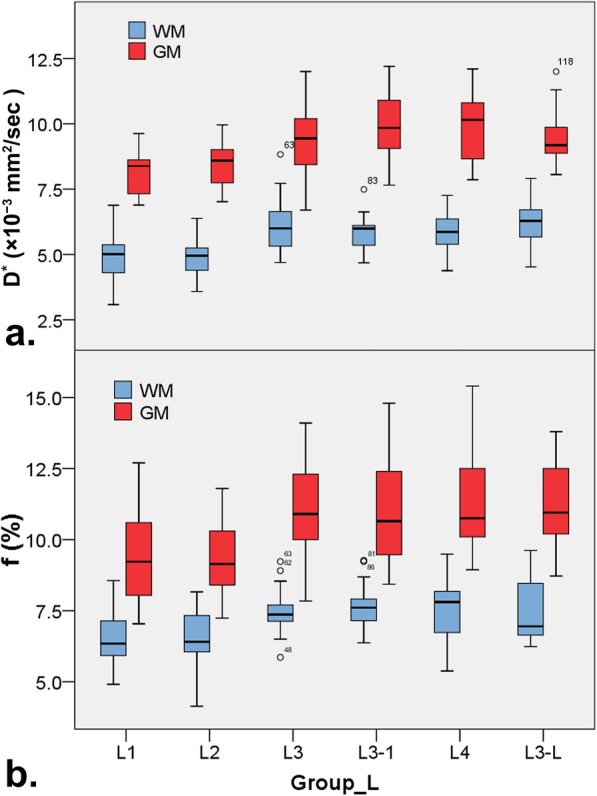
Box plots for pseudodiffusion parameter of D* (**a**) and f (**b**) derived from IVIM DWI among groups with different distribution of low b-value and NEX in brain white matter (WM) and gray matter (GM)

The descriptive statistics of the D, D* and f value of WM or GM among groups of low b-value distribution are shown in Table 2. The D* and f value of WM or GM in group L1 and L2 were significantly lower than ones in any other group L3, L4 and L3-L (all *P* <  0.05), but no difference exists between group L1 and L2, among L3, L4, or L3-L (all *P* > 0.05) (D*, 5.05, 4.93, 6.11, 5.84 and 6.24 × 10^− 3^ mm^2^/s in WM, and 8.18, 8.38, 9.49, 9.79 and 9.49 × 10^− 3^ mm^2^/s in GM; f, 6.56, 6.51, 7.48, 7.64 and 7.53% in WM, and 9.39, 9.26, 11.07, 11.03 and 11.31% in GM, in group L1, L2, L3, L4 and L3-L, respectively, as shown in Fig. 4 and Table 2). There were no differences of D value in WM or GM among each group (all *P* > 0.05) (as shown in Table 2).

**Table 2:** Compared the IVIM parameter derived from multiple b-value DWI among groups with different low b-value distribution and NEX in brain

Groups	D (× 10^− 3^ mm^2^/s)	D^*^ (× 10^− 3^ mm^2^s)	f (%)
White matter			
L1	0.714 ± 0.028	5.050 ± 0.887^a^	6.558 ± 0.900^a^
L2	0.718 ± 0.034	4.934 ± 0.777^a^	6.505 ± 0.991^a^
L3	0.716 ± 0.034	6.105 ± 0.996^b^	7.484 ± 0.766^b^
L4	0.718 ± 0.031	5.841 ± 0.645^b^	7.643 ± 0.813^b^
L3_1	0.718 ± 0.033	5.817 ± 0.780^b^	7.550 ± 1.007^b^
L3_L	0.718 ± 0.031	6.241 ± 0.734^b^	7.533 ± 1.112^b^
Gray matter			
L1	0.806 ± 0.039	8.180 ± 0.895^a^	9.394 ± 1.569^a^
L2	0.807 ± 0.037	8.376 ± 0.874^a^	9.259 ± 1.233^a^
L3	0.840 ± 0.041	9.487 ± 1.317^b^	11.066 ± 1.517^b^
L4	0.841 ± 0.054	9.794 ± 1.305^b^	11.025 ± 1.926^b^
L3_1	0.833 ± 0.029	9.854 ± 1.291^b^	11.303 ± 1.602^b^
L3_L	0.836 ± 0.051	9.496 ± 1.045^b^	11.308 ± 1.285^b^

Values marked with different letters have significant differences between any two groups both in white matter and gray matter (*P* <  0.05)

### NEX for low b value did not affect metrics of IVIM-DWI in brain

There was no difference in the D, D* and f value of WM or GM between group L3 and L3–1 (D, 0.716 and 0.718 × 10^− 3^ mm^2^/s; D*, 6.105 and 5.817 × 10^− 3^ mm^2^/s; f, 7.484 and 7.550% in group L3 and L3–1, respectively, *P* > 0.05) (as shown in Fig. 4 and Table 2).

### Parameters comparisons among different groups of high b-value distribution

**Fig. 5:**
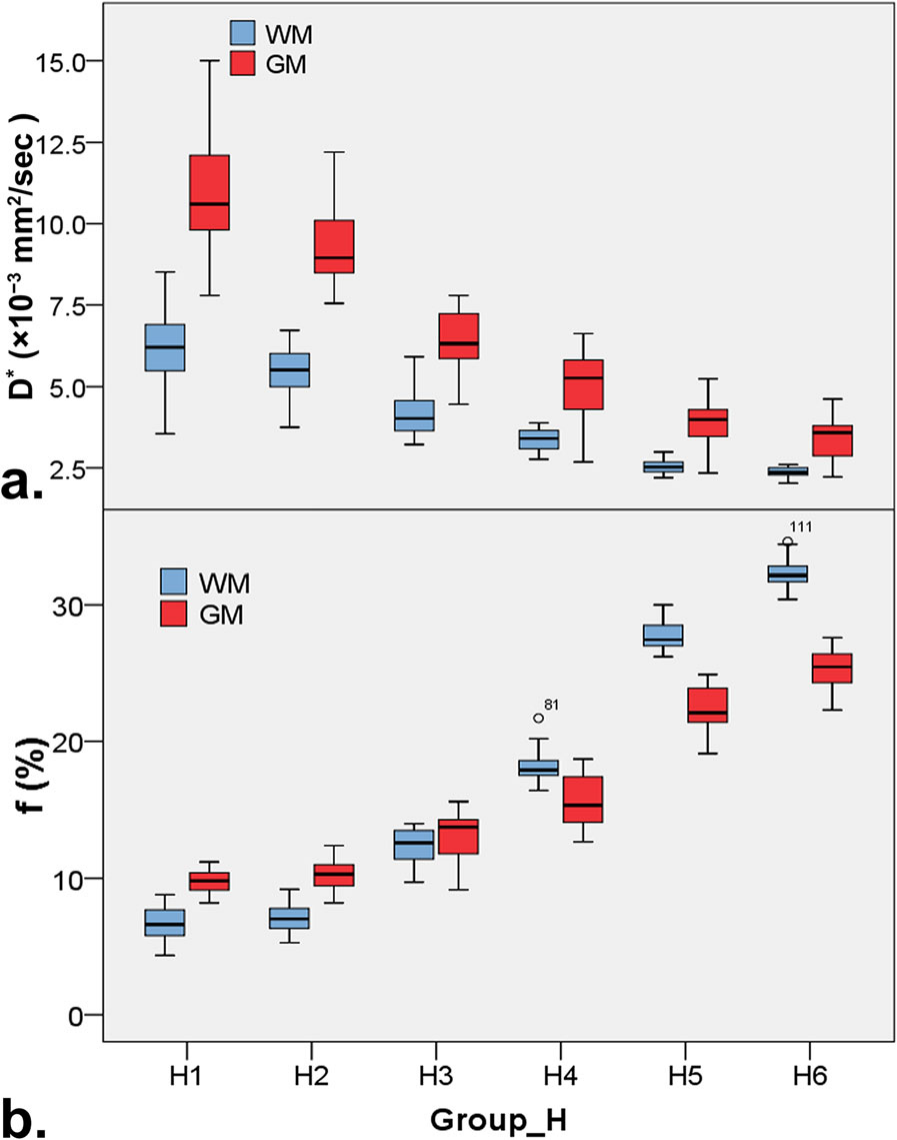
Box plots for pseudodiffusion parameter of D* (**a**) and f (**b**) derived from IVIM DWI among groups with different high b-value upper limit in brain white matter (WM) and gray matter (GM)

The descriptive statistics of the D, D* and f value of WM or GM among groups of high b-value distribution are shown in Table 3. The D and D* value both in WM and GM were significantly decreased with the increase of high b-value distribution except D and D* value between group H1 and H2 both in WM and GM, and D* value between group H5 and H6 in GM (D, 0.740, 0.718, 0.647, 0.579, 0.481 and 0.432 × 10^− 3^ mm^2^/s in WM, and 0.868, 0.839, 0.758, 0.712, 0.651 and 0.620 × 10^− 3^ mm^2^/s in GM; D*, 6.199, 5.450, 4.150, 3.379, 2.533 and 2.365 × 10^− 3^ mm^2^/s in WM, and 11.027, 9.264, 6.312, 4.986, 3.869 and 3.377 × 10^− 3^ mm^2^/s in GM, in group H1, H2, H3, H4, H5 and H6, respectively, as shown in Fig. 5a and Table 3).

**Table 3:** Compared the IVIM DWI parameter among groups with different high b-value upper limit in brain

Groups	D (×10^−3^ mm^2^/s)	D^*^ (× 10^− 3^ mm^2^/s)	f (%)
White matter			
H1	0.740 ± 0.034^a^	6.199 ± 1.223^a^	6.595 ± 1.188^a^
H2	0.718 ± 0.029^a^	5.450 ± 0.780^a^	7.098 ± 1.073^a^
H3	0.647 ± 0.021^b^	4.150 ± 0.694^b^	12.381 ± 1.200^b^
H4	0.579 ± 0.032^c^	3.379 ± 0.322^c^	18.055 ± 1.220^c^
H5	0.481 ± 0.035^d^	2.533 ± 0.198^d^	27.827 ± 1.099^d^
H6	0.432 ± 0.038^e^	2.365 ± 0.141^e^	32.241 ± 1.161^e^
Gray matter			
H1	0.868 ± 0.034^a^	11.027 ± 1.836^a^	9.781 ± 0.871^a^
H2	0.839 ± 0.032^b^	9.264 ± 1.215^b^	10.225 ± 1.180^a^
H3	0.758 ± 0.023^c^	6.312 ± 1.000^c^	13.200 ± 1.759^b^
H4	0.712 ± 0.024^d^	4.986 ± 1.017^d^	15.664 ± 1.760^c^
H5	0.651 ± 0.026^e^	3.869 ± 0.748^e^	22.445 ± 1.563^d^
H6	0.620 ± 0.035^f^	3.377 ± 0.637^e^	25.277 ± 1.499^e^

Values marked with different letters have significant difference between any two groups both in white matter and gray matter (*P* <  0.05)

The f value was significantly increased with the increase of high b-value distribution, but no significant difference was found between group H1 and H2 both in WM and GM (f, 6.595, 7.098, 12.381, 18.055, 27.827 and 32.241% in WM, and 9.781, 10.225, 13.200, 15.664, 22.445 and 25.277% in GM, in group H1, H2, H3, H4, H5 and H6, respectively, as shown in Fig. 5b and Table 3). In addition, f value in GM were higher than ones in WM both in group H1 and H2, while no significant difference was found in group H3, and inversed results obtained in group H4, H5 and H6 (as shown in Fig. 5b and Table 4).

**Table 4:** Compared the pseudodiffusion parameter derived from IVIM DWI between normal WM and GM among groups with different high b-value distribution

Groups	White matter	Gray matter	t	P
D^*^ (×10^−3^ mm^2^/s)				
H1	6.199 ± 1.223	11.027 ± 1.836	10.267	< 0.001
H2	5.450 ± 0.780	9.264 ± 1.215	12.392	< 0.001
H3	4.150 ± 0.694	6.312 ± 1.000	8.330	< 0.001
H4	3.379 ± 0.322	4.986 ± 1.017	7.066	< 0.001
H5	2.533 ± 0.198	3.869 ± 0.748	8.101	< 0.001
H6	2.365 ± 0.141	3.377 ± 0.637	7.273	< 0.001
f (%)				
H1	6.595 ± 1.188	9.781 ± 0.871	10.147	< 0.001
H2	7.098 ± 1.073	10.225 ± 1.180	9.193	< 0.001
H3	12.381 ± 1.200	13.200 ± 1.759	1.804	0.078^a^
H4	18.055 ± 1.220	15.664 ± 1.760	5.237	< 0.001
H5	27.827 ± 1.099	22.445 ± 1.563	13.210	< 0.001
H6	32.241 ± 1.161	25.277 ± 1.499	17.231	< 0.001

^a^ indicates no significant difference between groups (*P* > 0.05)

In addition, as shown in Fig. 3, the scattered red color area representing high pseudodiffusion effect in brain tissue was reduced with the increase of high b-value upper limit on pseudodiffusion images, indicating the attenuation of fast diffusion effect.

## Discussion

In this study, the influence of low b-values distribution, high b-values upper limit, and NEX for low b-values on IVIM parameters was investigated by in vivo brain multi-b-value DWI. The results of this study indicated that the D* and f value derived from IVIM DWI could be affected when there were less b-values in low b-value distribution (0 < b < 200 s/mm^2^). We also identified that the NEX for low b-values might not affect the IVIM metrics in brain. In addition, this study demonstrated significant differences in D, D* and f values among different distributions of high b-value derived from brain IVIM DWI of healthy volunteers.

IVIM DWI was found to be a valid and promising method to quantify water molecule diffusion and microvascular perfusion of living tissue perfusion, experiencing a remarkable revival for applications throughout the body, especially for oncologic patients, allowing earlier detection, diagnosis, staging, and monitoring of disease progression or response to therapy [24, 29, 45, 46]. Previously published studies of IVIM imaging have shown that the amount of low b-values significantly influence the pseudodiffusion metrics [41, 45]. Since the IVIM effect is usually small, more images or b-values are often acquired at low b-values than for diffusion at high b values [29]. In this study, D* and f value of WM or GM in more low b-values (more than eight b-values) were significantly larger than that in less low b-values distribution (less than five b-values). An in vivo liver MRI results in the normal controls revealed that the measured pseudodiffusion value was lower when calculated with less low b values (no b values between 0 and 50 s/mm^2^) compared to when calculated with more low b value (extra b = 10 and 25 s/mm^2^ were included) in the low b-value distribution [41], which were consistent with current research findings. Moreover, consistent with a recent study [47], there was no significant difference of the mean D* and f value among groups using eight or eleven low b-values distribution. The results indicated that insufficient numbers of low b-values might underestimate pseudodiffusion metrics. Compared with eleven b values, eight low b values (0, 30, 60, 90, 120, 150, 180 and 200 s/mm^2^) can significantly reduce scan time and may be sufficient for IVIM measurements.

The higher NEX was very important for high b-values to obtain higher signal to noise ratio (SNR) DW images, but it is still controversial whether the higher NEX for low b-values is necessary for calculating pseudodiffusion parameters more accurately in brain [21, 32, 41]. A main body of previous literature suggested that more images of the low b-values less than 200 s/mm^2^ were required for the calculation of pseudodiffusion, so higher NEX for very low b-values than for the medium b-values should be adopted in IVIM studies [31, 41]. However, we noticed that there was no significant difference of D* and f value derived from IVIM sequence using the same b-values and different NEX in current study. Although it has been confirmed that separation of perfusion from diffusion requires good SNR [21], the NEX for low b value may be less important since the SNR is higher at low b-values in brain. In this study, echo planar imaging (EPI) sequence with multi-channel receive coil and array spacial sensitivity encoding technique (ASSET) was adopted, so the current SNR of low b value DW image with low NEX might enough to satisfy the need of pseudodiffusion measurement [32], and the effect of NEX at low b values on IVIM parameters can be considered as negligible.

In the present study, we also evaluated how the upper limit for the high b-value distribution influences the in vivo IVIM measurements. We detected significant differences in parameters D, D* and f value among majority of high b-value distribution groups both in WM and GM. As the upper limit for the high b-value distribution increased, the D and D* values decreased and f value increased significantly. As a matter of fact, non-Gaussian diffusion becomes visible when high b values are used, and the degree of diffusion related signal attenuation or apparent diffusion coefficient decreases when the high b value increases [29]. It is, thus, mandatory to indicate that D value decreases with the b value increases according to the IVIM model eq. (2), and correspondingly, an accurate D* and f value can be obtained when high b-value upper limit at less than 1000 s/mm^2^. However, by using such high b values (b value larger than 1500 s/mm^2^), the IVIM model reaches its limitation, as it cannot give a proper perfusion measurement [29], thus the decreased D value may lead to a biased D* and f value. This phenomenon may explain the contradictory results in many IVIM studies. For example, the f values were significant higher in HGG than in LGG using relatively reasonable upper limit for the high b-value distribution (b value less than 1000 s/mm^2^) in some studies [37, 40], but other studies using higher upper limit for the high b-value distribution (b value larger than 1500 s/mm^2^) got inconsistent results in that the f values were significantly lower in HGG than in LGG group [22, 38, 39]. Also demonstrated in current study, the f values were significant lower in WM than in GM when using a high b-value upper limit of less than 1000 s/mm^2^, but converse results were demonstrated when using a high b-value upper limit higher than 1500 s/mm^2^. In addition, at high b values, because of the nature of the MR imaging signal (a magnitude signal that cannot be negative), there is always some background noise signal left [29], which may affect IVIM parameter metrics. In summary, DWI model when b-values larger than 1500 s/mm^2^ is a non-linear fitting curve (diffusion is non-Gaussian), more elements of kurtosis imaging rather than IVIM, we cannot obtain accurate pseudodiffusion metrics by using bi-exponential equation.

It is well known that blood flow in randomly oriented capillaries (at voxel level) mimics a random walk, which results in a pseudodiffusion effect in the presence of diffusion encoding gradient pulses. Indeed, the IVIM imaging has a differential sensitivity to vessel sizes, according to the range of b values that are used [29]. Similarly, in our study, the red color area representing significant pseudodiffusion effect within the brain tissue was reduced with the increase of high b-value upper limit on pseudodiffusion images, indicating the attenuation of fast diffusion effect derived from blood flow in small vessel or cerebrospinal fluid (CSF) space [48]. We noticed that the red color area or fast diffusion effect was relative absent in brain tissue on pseudodiffusion images using higher upper limit for the high b-value distribution (b value higher than 1500 s/mm^2^). But when a rather low b-value upper limit, for example less than 500 s/mm^2^, was adopted, considerable partial volume from marked IVIM effect of small vessel and CSF space could influence the pseudiffusion measurement derived from brain micro circulation [48]. Thus, from this point of view, we recommend a proper high b-value upper limit (800–1000 s/mm^2^) as the referred standard for brain IVIM DWI studies.

One important consideration of using the IVIM technique based on DW MRI is obtaining reliable and repeatable data from patients. In our study, to ensure data accuracy or consistency, we obtained IVIM parameters from contralateral frontal lobe in each participant. Analysis revealed no significant difference of IVIM parameters between two measurements in right and left frontal lobe, which suggested the reliability of the obtained data in our study.

Our study has some limitations. First, no simulation experiments were performed to look at the effects of low b-values on IVIM calculations before in vivo MRI, however, our designs of the b-value selection based on the previous simulation and in vivo research [36, 41, 45, 49]. Second, food intake was not controlled, food intake could potentially influence brain perfusion, and however, pseudodiffusion parameters comparisons were made between different b-value combinations within the same scans and not between subjects, these effects should be minimal in this study. Thirdly, only two-step fitting method was used in this study, another fitting model such as one-step direct fitting technique or Bayesian fitting was not assessed. Comparing with one-step direct fitting technique, the segmented IVIM fitting method was used to increase robustness under biological conditions. Bayesian modeling is capable of producing more visually pleasing IVIM parameter maps than least squares approaches, but their potential to mask certain tissue features demands caution during implementation [50]. Finally, in the current study, hand-drawn ROIs used for each group were not absolutely identical, which may lead to a sampling bias.

## Conclusion

This study demonstrated that D* and f value derived from in vivo brain IVIM-DWI could be affected when there were less low b-values distribution (0 < b < 200 s/mm^2^). Eight low b-values with upper limit for the high b-value distribution of 800–1000 s/mm^2^ might be the relatively proper set when performing brain IVIM-DWI. We also identified that the NEX selection for low b-value does not influence the brain IVIM metrics.

## Data Availability

The datasets analyzed during the current study are not publicly available. These data could only be accessed to researchers to ensure participant confidentiality.

## Abbreviations

ANOVA: Analysis of variance
ASSET: Array spacial sensitivity encoding technique
CSF: Cerebrospinal fluid
D: Slow diffusion coefficient
D*: Fast perfusion coefficient
DWI: Diffusion-weighted imaging
EPI: Echo planar imaging
f: Perfusion fraction
FLAIR: Fluid-attenuated inversion recovery
FOV: Field of view
GM: Gray matter
HGG: High grade glioma
IVIM: Intravoxel incoherent motion
LGG: Low grade glioma
MRI: Magnetic resonance imaging
NEX: Number of excitation
ROI: Region of interest
SNR: Signal to noise ratio
TE: Echo time
TR: Repetition time
WM: White matter
